# Supplementary material for: Chemoreceptor co-expression in Drosophila melanogaster olfactory neurons
Source: eLife. 2022 Apr 20;11:e72599. doi: 10.7554/eLife.72599 (PMC9020824; doi:10.7554/eLife.72599)

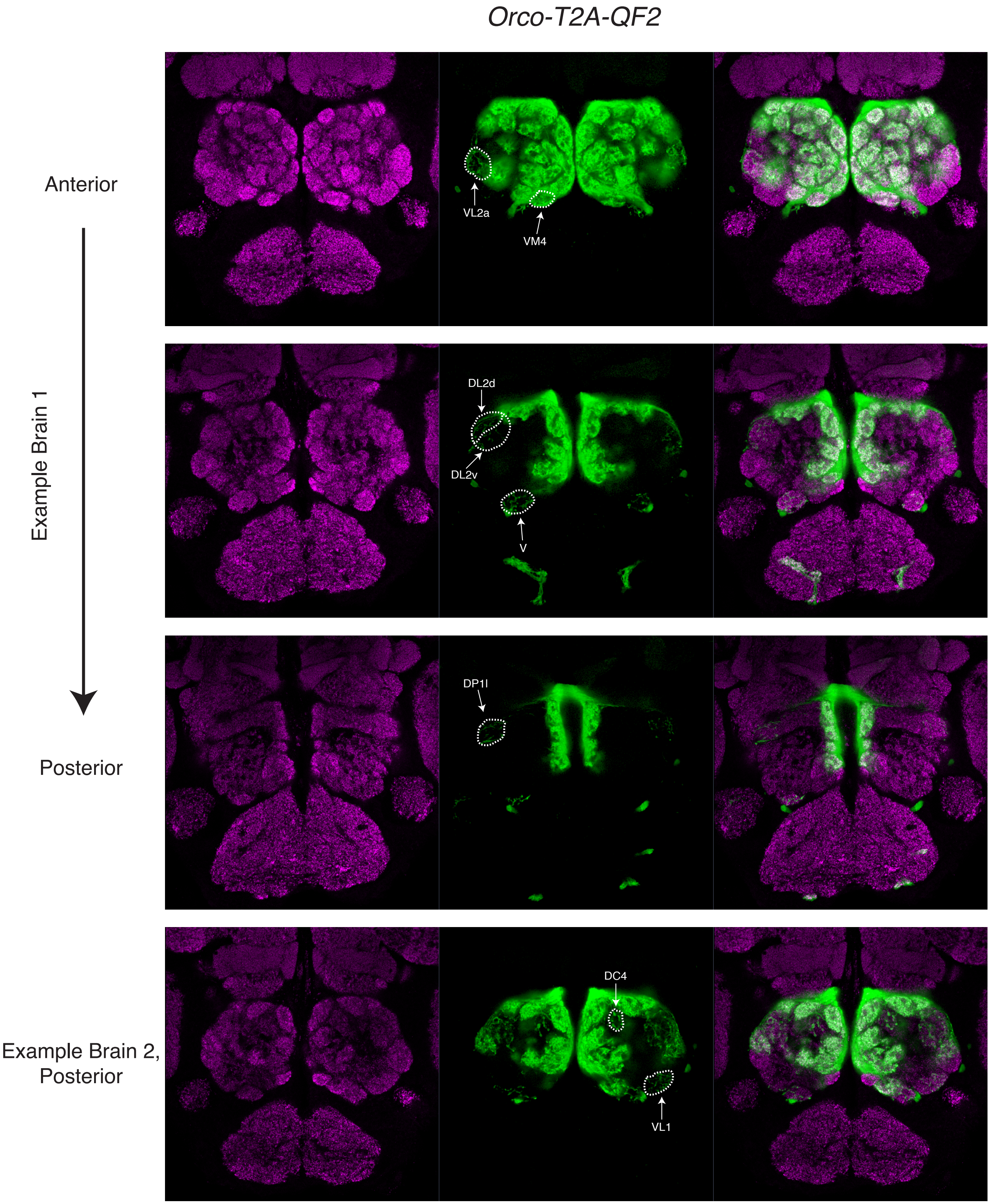
Orco-T2A-QF2
Anterior
Example Brain 1
Posterior
Example Brain 2, Posterior
VL2a
VM4
DL2d
DL2v
V
DP1l
DC4
VL1

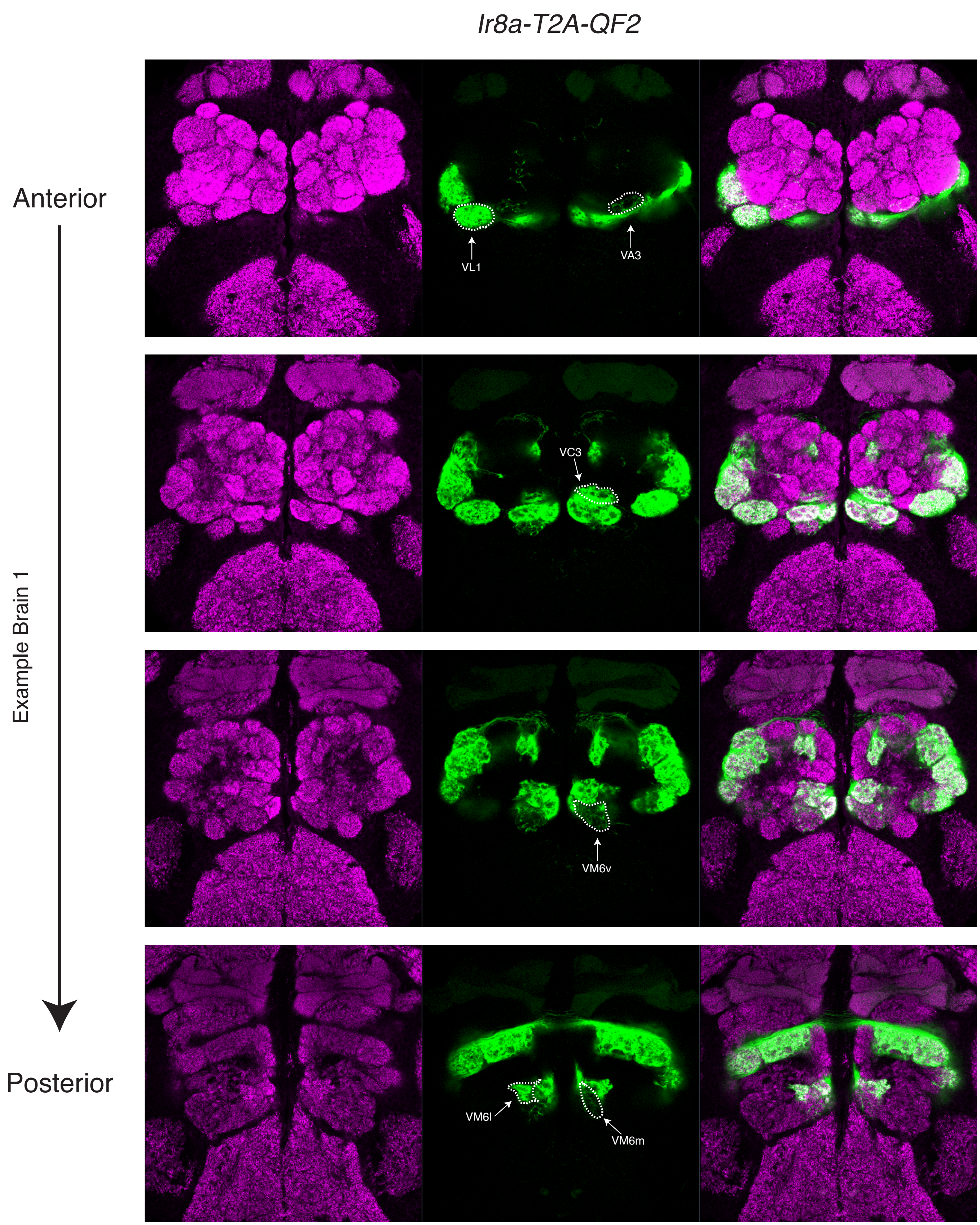

Ir8a-T2A-QF2
Anterior
Example Brain 1
Posterior
VL1
VA3
VC3
VM6v
VM6l
VM6m

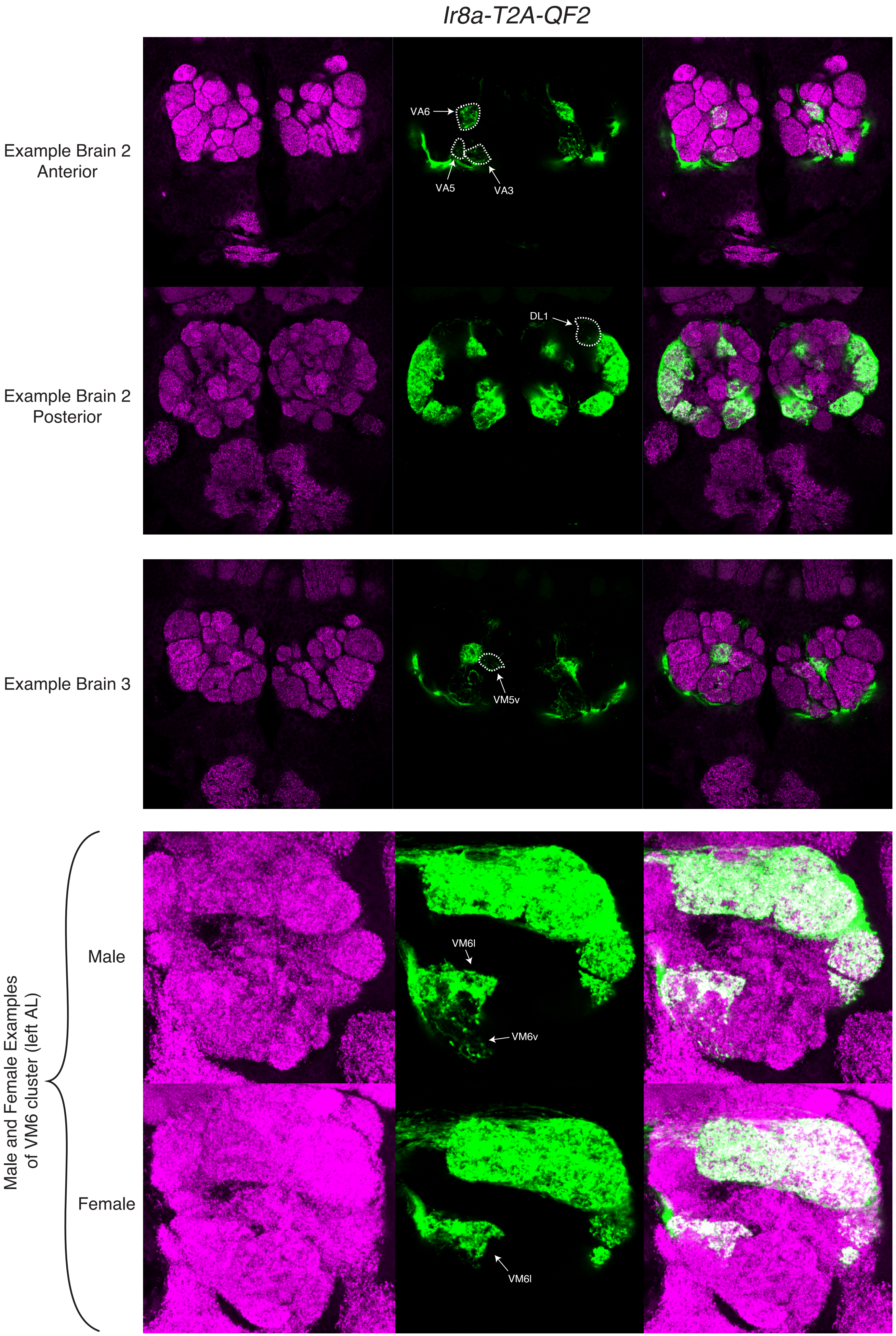

Ir8a-T2A-QF2
Example Brain 2
Anterior
VA6
VA5
VA3
Example Brain 2
Posterior
DL1
Example Brain 3
VM5v
Male and Female Examples
of VM6 cluster (left AL)
Male
VM6l
VM6v
Female
VM6l

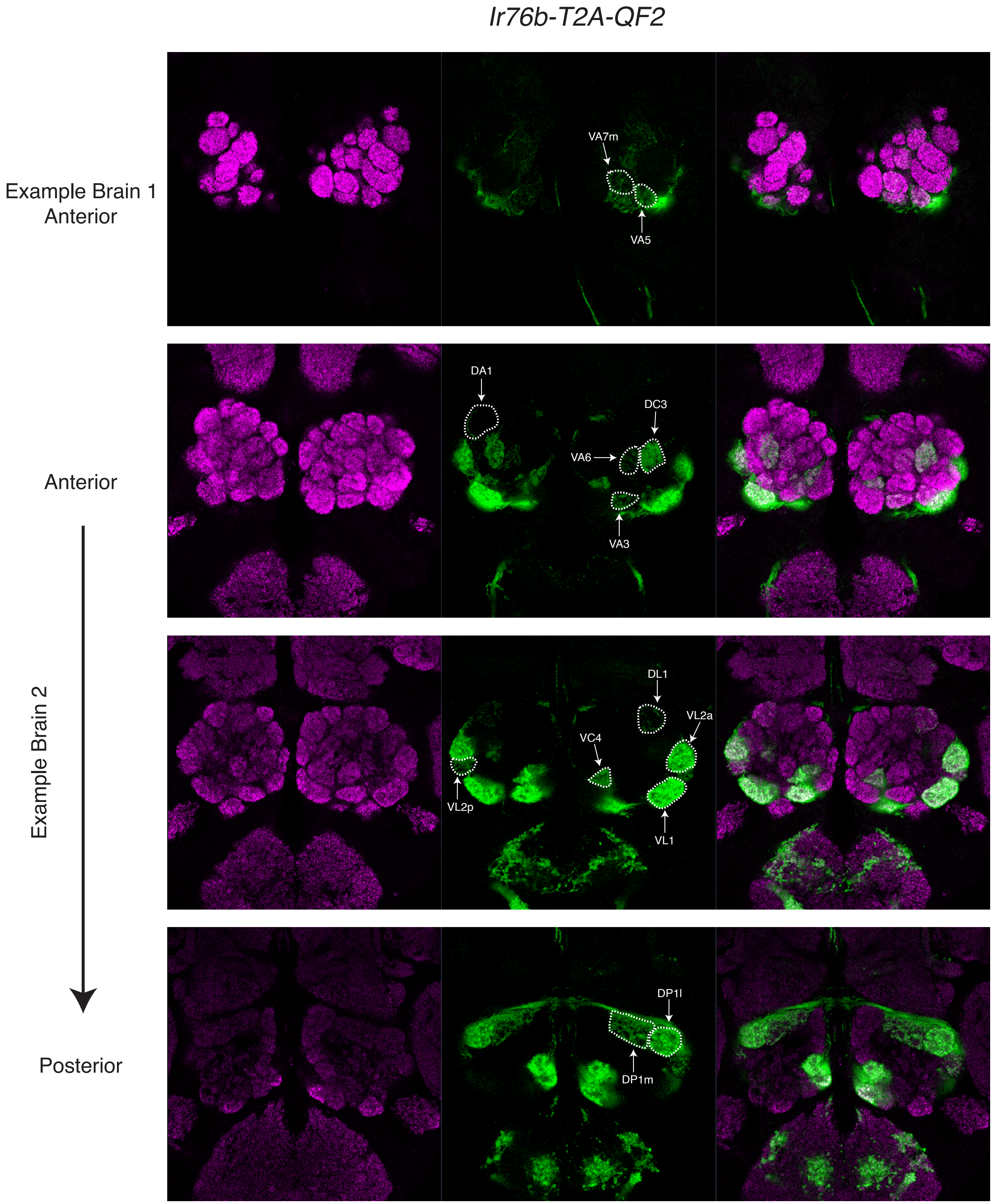

Ir76b-T2A-QF2
Example Brain 1
Anterior
VA7m
VA5
DA1
DC3
VA6
VA3
Anterior
Example Brain 2
DL1
VL2a
VC4
VL2p
VL1
DP1l
DP1m
Posterior

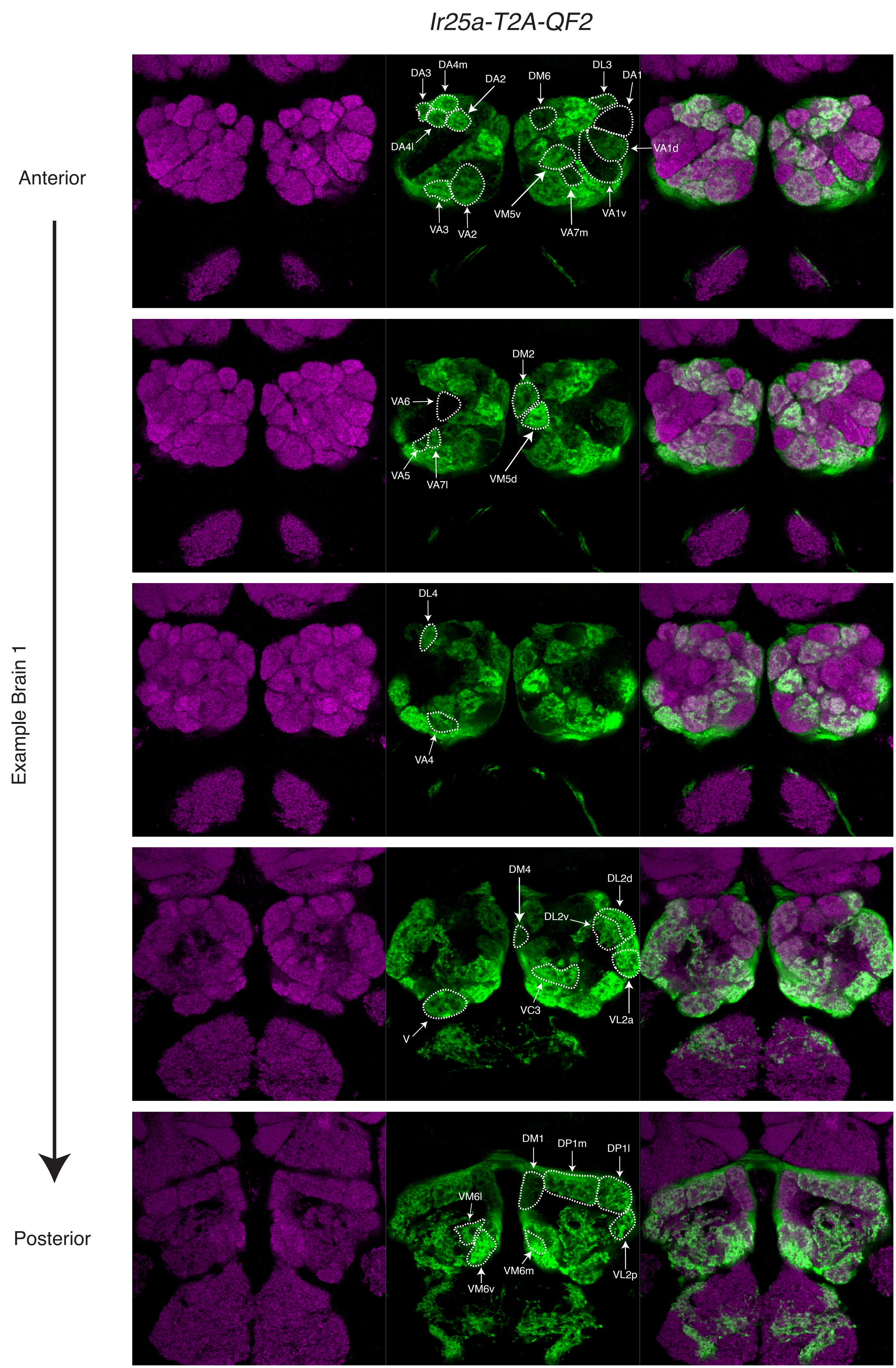

Ir25a-T2A-QF2
Anterior
Example Brain 1
Posterior
DA3
DA4m
DA2
DM6
DL3
DA1
DA4l
VA1d
VM5v
VA7m
VA1v
VA3
VA2
DM2
VA6
VA5
VA7l
VM5d
DL4
VA4
DM4
DL2d
DL2v
VC3
VL2a
V
DM1
DP1m
DP1l
VM6l
VM6m
VM6v
VL2p

Ir25a-T2A-QF2

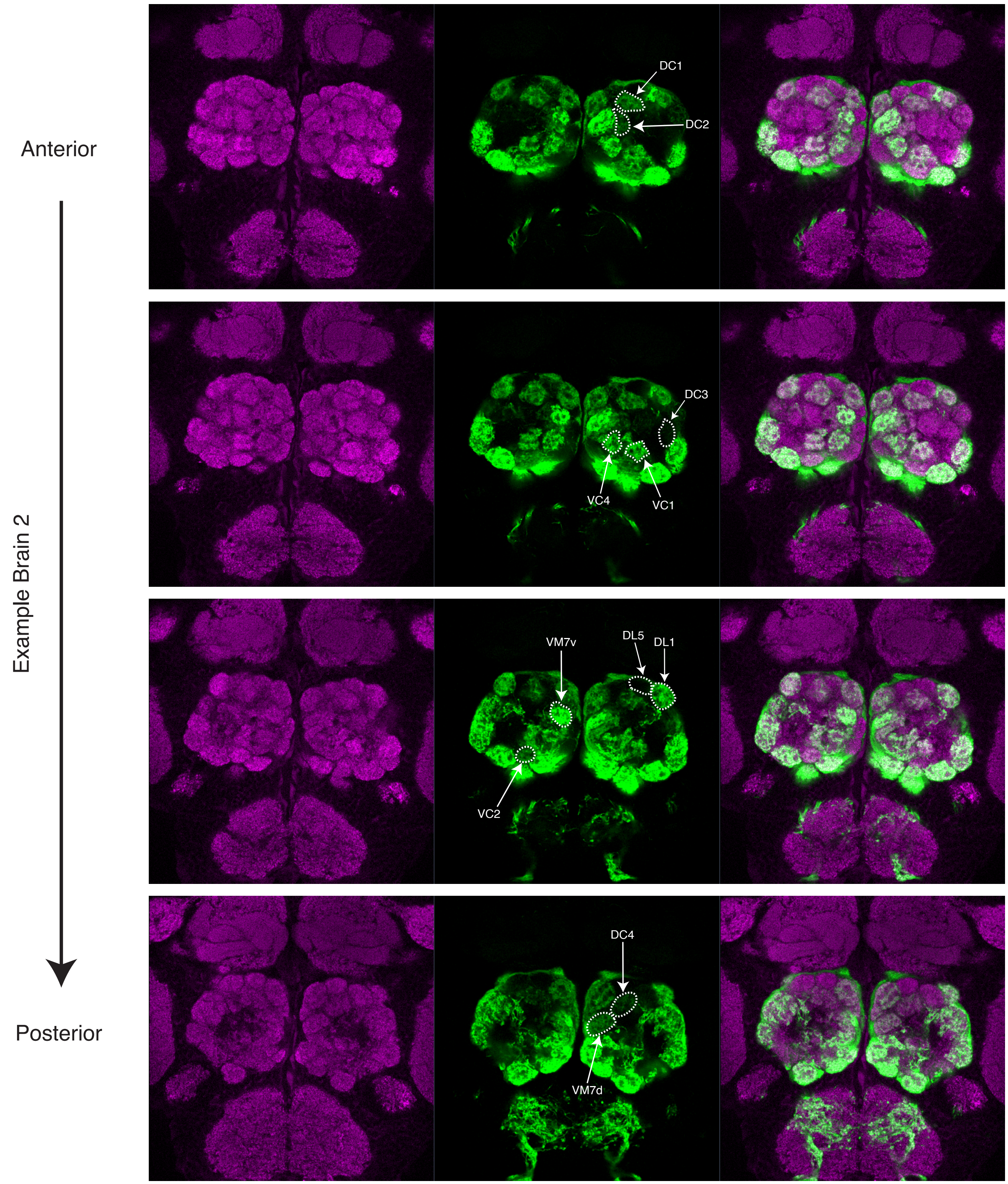

Supplement: Figure 3—source data 2. [file elife-72599-fig3-data2.pdf]
